# Veterinary Bath

**Published:** 1818

**Authors:** James Carver

**Affiliations:** V. S.; Philadelphia


### VETERINARY BATH.

*To Private Gentlemen, Owners of Breweries, Proprietors of Mail Stage Establishments, and Livery Stable Keepers, the following Improvement in Veterinary Science, is respectfully submitted.*

THE various uses to which Dr. Carver's *Patent Universal Veterinary Medical Bath* may be applied, particularly for restoring the feet of horses that have been injured by constant use on the pavement of a large city, renders it a valuable *desideratum* to the public. The principles of this Bath, and the various medical uses to which it may be applied, in diseases of the feet, such as founder, contraction, and sand-cracks—of the skin—of the lungs—and for inflammation generally; for tetanus, and many other disorders, both external and internal, to which horses and other quadrupeds are liable, makes it of vast importance. It is also recommended as useful in all infirmaries and large establishments, such as breweries, sugar houses, mail stage establishments, private and public stables, &c. To the southward and westward, and in the West Indies, on large and extensive cotton farms, and plantations, where tetanus and other spasmodic disorders so frequently occur among the negroes, the hot and vapour bath will be found extensively useful in saving the life of both man and beast. Dr. Carver, with a view of making its usefulness more general, has simplified his Universal Bath, so as to render it less expensive, as well also to give less trouble and inconvenience to servants.

A model of Dr. Carver's Universal Bath may be seen at his residence; and all applications for the right of using it, together with the Patent Bar Shoe, in aid of the cure of Contraction, will be punctually attended to.

For the above mentioned establishments, the following charges will be made, for patent rights for the term of *fourteen years:*

| | |
|---|---|
| For all public livery stables, . . | 10 dolls. |
| Mail stage establishments, . . . | 10 |
| Breweries, not exceeding ten horses, . . | 5 |
| Private stables, do. do. . . | 5 |
| Private gentlemen, owning only one horse, . | 3 |

All applications for the purchase of patent rights for a town, city or stable, will be punctually attended to.

J. CARVER, *V. S.*

Philadelphia, August, 5, 1818.

